# Interstitial Lung Disease: Does It Represent a Real Comorbidity in Spondyloarthritis Patients? Results from an Ultrasound Monocentric Pilot Study

**DOI:** 10.3390/jcm14165632

**Published:** 2025-08-09

**Authors:** Andrea Delle Sedie, Linda Carli, Annamaria Varrecchia, Cosimo Cigolini, Marco Di Battista, Lorenzo Esti, Federico Fattorini, Emanuele Calabresi, Marta Mosca

**Affiliations:** 1 Rheumatology Unit, University of Pisa, 56126 Pisa, Italy; 81clinda@gmail.com (L.C.); cosimo.cigolini@gmail.com (C.C.); dibattista.marco91@gmail.com (M.D.B.); lorenzo.esti@gmail.com (L.E.); f.fattorini6@studenti.unipi.it (F.F.); marta.mosca@unipi.it (M.M.); 2 Department of Medical and Surgical Sciences, University of Bologna, 40126 Bologna, Italy; annavarrecchia@outlook.it; 3 Rheumatology Unit, San Donato Hospital, 52100 Arezzo, Italy; emanuele.calabresi@uslsudest.toscana.it

**Keywords:** interstitial lung disease, spondylarthritis, psoriatic arthritis, ankylosing spondylitis, ultrasound, lung

## Abstract

**Background/Objectives:** Interstitial lung disease (ILD) is a frequent complication of rheumatoid arthritis (RA), representing the most common extra-articular manifestation (with a prevalence of about 10–60%) and the second cause of mortality. Spondyloarthritides (SpAs) are chronic arthritides that share with RA both a similar disease burden and similar therapeutical approaches. The evaluation of ILD is challenging, given the low sensitivity of X-ray and pulmonary function tests, and the radiation exposure linked to repetitive HRCT. Lung ultrasound (LUS) has shown potential in the evaluation of ILD in autoimmune diseases. The purpose of this study is to assess the prevalence of ILD in a cohort of SpA patients (pts) using LUS in comparison with healthy subjects (HSs). The secondary aim is to evaluate potential correlations between ILD and clinical features within the SpA cohort using LUS. **Methods:** Consecutive SpA out-patients were examined by LUS, applying the definition for pleural line irregularity (PLI) recently provided by the OMERACT taskforce for LUS in systemic sclerosis. Seventy-one intercostal spaces were studied (14 in the anterior chest, 27 lateral and 30 posterior) in all the pts/HS using an Esaote MyLab25 Gold US machine with a linear 7.5–10 MHz probe. A total pleural score was calculated. Each patient answered to Italian-validated PROs on respiratory function (Leicester and Saint-George), global health (SF-36) and dyspnea (mMRC scale). Clinical data on disease duration, disease onset, disease activity (at the moment of the examination) and methotrexate (MTX) or biologics treatment were collected from the medical records. **Results:** Seventy-three SpA pts (46 psoriatic arthritis -PsA- and 27 ankylosing spondylitis -AS-) and 56 HS were studied. No significant differences were demonstrated between groups (SpA vs. HS and PsA vs. AS) for age, sex, BMI and smoking habits. The total PLI score was significantly higher in SpA pts than in HS (*p* < 0.001). A positive correlation was found between the total PLI score and the PLI score from anterior, posterior and lateral chest. The posterior region of the chest showed a higher PLI score than the anterior and lateral regions. No statistically significant differences were found between PsA and AS. MTX use was not a risk factor for PLI (no differences were found between SpA MTX+ and SpA MTX- patients). PROs (Leicester, Saint-George and SF-36) were not related to the PLI total score. A significant correlation was found only between the SF36 score and the presence of PLI in the anterior chest. PROs were instead correlated with each other, showing a good concordance for absence/presence of symptoms. Disease activity, disease duration and age at disease-onset were not related to PLI total score. Smoking habit was found to be predictive of a significantly higher PLI score both in SpA patients and HSs. **Conclusions:** LUS examination shows a higher amount of PLI in SpA patients with respect to HSs. Smoking habit was the only clinical feature correlated to PLI on LUS examination in our population.

## 1. Introduction

Recent evidence from the literature underlined how the lung could be considered as a “target” organ in systemic autoimmune diseases, in particular systemic sclerosis (SSc), idiopathic inflammatory myopathies (IIMs), primary Sjogren syndrome and rheumatoid arthritis (RA) [1,2]. It tends to manifest itself more frequently as an interstitial lung disease (ILD), than as an inflammatory involvement of the lung, with specific histologic and radiologic patterns, with different clinical presentation and prognosis. While inflammatory cell infiltrates are the predominant feature in nonspecific interstitial pneumonia (NSIP), fibrosis represents the main histopathological component in usual interstitial pneumonia (UIP) [3]. ILD, usually presenting with a UIP pattern, is a frequent complication in rheumatoid arthritis (RA) [4], representing the most common extra-articular manifestation (with a prevalence of about 10–60%), and the second cause of mortality (after cardiovascular diseases). Spondylarthritis (SpA) is a chronic form of arthritis that shares with RA both a similar burden of disease (i.e., peripheral arthritis, age of patients, impact on the quality of life, etc.…), some similar cytokines pathways (i.e., the TNFa pathway) and similar therapeutical approaches (i.e., leflunomide, methotrexate and TNFa inhibitors). Lung involvement in SpA may occur at every stage of the disease and may present in ankylosing spondylitis (AS) patients as ILD, upper lobe fibrosis, emphysema, bronchiectasis and functional ventilation defects, as already shown in 2007 by Sampaio-Barros et al., who found high resolution computer tomography (HRCT) abnormalities in 21/52 patients, mostly nonspecific linear parenchymal opacities (19%), lymphadenopathy (12%), emphysema (10%), bronchiectasis (8%) and pleural involvement (8%) [5]. Its prevalence in AS varies according to the diagnostic tool used, ranging from 8% using conventional radiology to 40–80% using HRCT and 18–42% with pulmonary function tests (PFTs). El Maghraoui et al., in a recent systematic literature review (SLR), highlighted the discrepancy between the prevalence of symptomatic patients (5.9% out of 303) and the prevalence of ILD-related changes on the HRCT (61%) in a population of AS patients. The more common HRCT findings were pleural thickening (18%), parenchymal band (15%), interlobular thickening (11%) and ground-glass areas (11%). A correlation between disease duration and the presence of HRCT findings was demonstrated only for the upper lobe fibrosis (in the 7% of patients) [6].

In psoriasis patients, an increased risk for chronic obstructive pulmonary disease (COPD) (RR 1.45) was demonstrated [7]. More recently, a significant link between psoriasis and ILD was noted [8,9]. ILD was identified in 8/392 psoriatic patients (2%) examined with HRCT during the screening for beginning biologic DMARDs (bDMARD) treatment. Those patients were presenting more frequently bilateral ground-glass and/or irregular linear (reticular) opacity in the lower lung zone [8]. In another study, involving patients referred to an ILD clinic, psoriasis was found in the 4.7% (21/447) of patients, presenting an usual interstitial pneumonia (UIP) HRCT pattern in nine patients, a nonspecific interstitial pneumonia (NSIP) pattern in six patients, an organizing pneumonia pattern in four patients and an hypersensitivity pneumonitis pattern in two patients [9]. Therefore, it appears that the most common HRCT pattern in psoriasis is the UIP pattern, similar to the evidence for RA. Yue et al. recently provided genetic evidence supporting the hypothesis that PSA may be a contributory risk factor for ILD in psoriatic patients [10]. Considering that psoriasis is part of the broader concept of “psoriatic disease”, we speculate that the UIP pattern is the one we could find in PsA too, confirming the “similarity” between RA and psoriatic disease.

Peluso et al. demonstrated lung involvement in very few patients with axial psoriatic arthritis (PsA) [11]. Nodular or ground-glass in the upper lobes and reticular pattern and honeycombing in the lower lobes were demonstrated by Bargagli et al. as the predominant HRCT pattern in PsA patients [12], supporting the concept of a UIP pattern in PsA. More recently, Schäfer et al. demonstrated chest radiography pulmonary involvement in 37.0% of a group of patients affected by RA and PsA (50% and 22.7%, respectively, in RA and in PsA), with only 35.3% of them being symptomatic [13]. Similarly, Provan et al. noticed an ILD incidence per 1000 person-years of 1.9, 0.6 and 0.2 in RA, PsA (with no previous ILD who initiated a first-ever treatment with a bDMARD) and the control group, respectively. The PsA patients who developed ILD were older and had higher levels of ESR and patient VAS at baseline compared to those who did not present ILD [14]. Despite this evidence, there is still a lack of data about lung involvement in SpA, particularly focusing on ILD.

ILD evaluation is challenging, given the low sensitivity of conventional radiology and PFTs, and radiation risk, which limits repetitive HRCT. Lung ultrasound (LUS), developed in the last 20 years, is a radiation-free, inexpensive, repeatable and patient friendly imaging technique. Since 2008, it has shown potential in the evaluation of ILD in autoimmune diseases [15,16,17,18,19,20,21,22,23,24,25,26,27,28,29,30,31,32,33,34,35,36,37,38,39,40,41,42,43,44,45,46]. LUS is based on the change in the alveolar air/tissue ratio, with an increment for solid tissue (i.e., fibrosis, blood, edema), which allows the creation of US artifacts. Most of time, there is a thickening of interlobular septae (both for fibrosis or edema) that creates ultrasound findings (B-lines) and irregularities in the pleural line (PLI); these findings are the US demonstration of a change in the normal structure of the lung.

The most studied US finding is represented by the so-called B-lines, defined by the Outcome Measure in Rheumatology (OMERACT) as a vertical hyperechoic reverberation artifact that arises from the pleural line, extends to the bottom of the screen without fading and moves synchronously with lung sliding [27]. B-lines are well diffused in the cardiogenic lung edema, and this is the reason why LUS was primarily used by cardiologists and pneumologists. In more recent years, LUS has also found growing application in the rheumatic field [15,16,17,18,19,20,21,22,23,24,25,26,27,28,29,30,31,32,33,34,35,36,37,38,39,40,41,42,43,44,45,46]. Pleural line evaluation (irregularity, thickening or fragmentation) for ILD assessment was initially described by Reißig & Kroegel [47] (together with B-lines and sub-pleural changes), and pleural line irregularity (PLI) is now defined as a loss of regularity that may be associated with an increase in thickness (either focal, diffuse, linear or nodular) [27].

Recently, a SLR demonstrated the face and content validity of LUS for the evaluation of ILD, as well as its feasibility, in systemic sclerosis and by extension, in other rheumatic musculoskeletal diseases that may present ILD as part of their clinical manifestation [26].

Considering the increasing role of LUS as a diagnostic tool for lung involvement in rheumatologic diseases, as well as its safety, we thought this technique might be used for the evaluation of pleural line irregularities (PLIs) in patients with SpA. Hence, the primary aim of our study was to use LUS to assess the prevalence of ILD in a cohort of SpA patients compared to healthy subjects (HS). The secondary aim was to explore potential correlations between ILD and clinical features within the SpA cohort, as well as to investigate possible differences between AS and PsA patients.

## 2. Materials and Methods

### 2.1. Patients

This is a cross-sectional study performed in the out-patient SpA Clinic of the Rheumatology Unit (Azienda Ospedaliero-Universitaria Pisana) between June 2024 and January 2025. This study was approved by the local Ethical Committee. Patients fulfilling the ASAS or CASPAR classification criteria [48,49] were enrolled over two consecutive 3-month periods (without any previous selection) and compared to the HSs matched for sex and age, with no history of rheumatic diseases, no known lung problems and no familial relationships with rheumatic patients. Demographic and clinical data, including sex, age, BMI, disease duration, disease onset, disease activity at the moment of the examination (assessed using DAPSA and ASDAS scores), presence of psoriasis and treatment with synthetic DMARDs (sDMARDs) or bDMARDs were collected from the medical records. Smoking habits (presence/absence and number of pack/year), the presence of respiratory symptoms (i.e., cough in the last 8 weeks) and their possible causes (i.e., COPD, asthma, gastroesophageal reflux or ACE-inhibitors treatment) were investigated, along with previous exposure to pneumotoxic substances like asbestos, amiodarone or radiotherapy. The presence of respiratory symptoms already diagnosed as specific for a pulmonary disease different from ILD, or the exposition to respiratory toxic substances were considered exclusion criteria of the study.

Each patient filled in the Italian-validated PROs on respiratory function (Leicester Cough Questionnaire—LCQ—and Saint-George’s Respiratory Questionnaire—SGRQ), global health (Short Form-36 Health Survey—SF-36) and dyspnea (Modified British Medical Research Council Questionnaire—mMRC). LCQ is used to evaluate how cough impacts on patients’ quality of life [50]; SGRQ investigates the features and intensity of respiratory disorders, as well as their impact on patents’ life [51] and mMRC assesses the degree of physical effort that causes the symptom, being significantly correlated with respiratory function [52].

### 2.2. Lung Ultrasound Assessment

A seventy-two intercostal space scanning protocol was used (14 in the anterior, 27 in lateral and 30 in posterior chest, along parasternal, mid-clavicular, anterior and posterior axillary, para-vertebral and scapular angle lines in all the patients/HSs. Scans were performed using an Esaote MyLab25 Gold (Esaote, Genoa, Italy) US machine with a linear 6–18 MHz probe set at a frequency of 10 MHz and a gain of 28%. As the scanning method has not yet been standardized, we employed a protocol previously used in our studies. This same protocol was also applied in a published paper in which the scoring system we adopted was validated against HRCT [24].

Patients and HSs were examined in a seated position for postero–lateral intercostal spaces and in a supine position for the assessment of the antero–lateral spaces.

**Figure 1:**
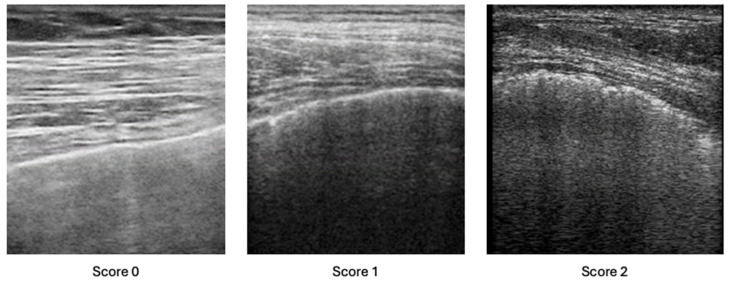
PLI scoring system.

Lung examination was made assessing only PLI and applying the definition recently provided by the OMERACT taskforce for LUS [27]. We used the scoring system by Pinal-Fernandez et al. [24], assigning a score of 0 if a normal pleural line was present, a score of 1 if pleural irregularity was moderate and a score of 2 if it was severe, as shown in Figure 1. To date, no cut-off score has been established for PLI positivity. Pinal-Fernandez et al. [24] showed that a cut-off of 24% resulted in a sensitivity of 79% and a specificity of 100% for the diagnosis of ILD, but this result was obtained in a population of patients with SSc and antisynthetase syndrome, with respect to the HRCT findings. As we applied LUS to a population with a lower expected ILD prevalence (without any HRCT data), we decided not to apply any cut-off value.

All examinations were performed by two different operators (C.E. and V.A.), who were previously trained by an expert ultrasonographer (D.S.A.) with more than 10 years of experience in LUS. Before the beginning of this study, intra- and inter- operator reproducibility was tested on video clips, using the same methodology applied to the OMERACT validation process [27]. We used a set of 30 high-quality video clips of pleural line irregularities obtained from systemic sclerosis patients, providing a balanced range of normal, low, medium and high degrees of lung disease involvement

### 2.3. Statistical Analysis

Continuous variables were reported as mean and standard deviation in case of parametric distribution and as median and interquartile range (IQR) for non-parametric distribution. Comparisons between group means were performed using the non-parametric Mann–Whitney test for the two groups and the Kruskal–Wallis test for comparisons involving three groups. Comparison of proportions was performed using the Chi^2^ test. The correlation between continuous variables was performed using Spearman’s test. A *p* < 0.05 was considered statistically significant. Fleiss’ kappa was used to assess the inter-rater agreement between sonographers for scoring PLI [53]. Reliability data were interpreted as follows: 0–0.20 represented slight agreement; 0.21–0.40, fair; 0.41–0.60, moderate; 0.61–0.80, good and >0.80, excellent reliability. All statistical analysis were performed with “R software”, version 4.4.2 (2025).

## 3. Results

Seventy-three SpA (46 with PsA and 27 with AS) patients and 56 HSs were enrolled. The demographic and clinical data are reported in Table 1. No significant differences were observed between groups (SpA vs. HS and PsA vs. AS) with respect to sex, age, BMI (numerically higher in PsA) and smoking habits (also including ex-smokers).

Disease activity, evaluated using ASDAS and DAPSA scores for AS and PsA, respectively, was higher in the AS group (the mean ASDAS score was 2.2 ± 1.4, indicating high disease activity, while the DAPSA score was 14.1 ± 10.5, indicating moderate disease activity).

Psoriasis (assessed by BSA) was present only in the PsA group (42/46 patients), with 22/42 showing skin involvement at the time of the LUS examination. All PsA patients with skin disease presented low levels of psoriasis (BSA < 5).

csDMARDs were prescribed significantly more often in PsA than in AS (23/46 vs. 7/27), with MTX being the most frequently used (MTX in 18, leflunomide in 7, hydroxicloroquine in 3 and sulfasalazine in 2 patients). MTX was predominantly used in PsA patients; however, a few AS patients were also treated with MTX due to peripheral joint involvement.

bDMARDs were more frequently used in AS. TNFa inhibitors were significantly more commonly prescribed in AS, whereas biologic therapies with alternative mechanisms of action were more frequently used in PsA.

### 3.1. Ultrasound Results

Inter-reader reliability was good (k = 0.77).

**Figure 2:**
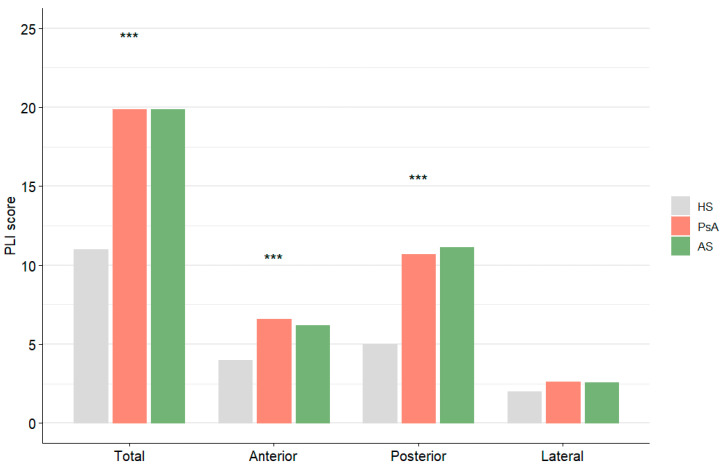
PLI score for different areas in HS, PsA and AS groups. *** *p* < 0.001.

The mean PLI score in patients (AS + PsA) was 19.9 ± 10.9 (range 0–51) with a statistically significant difference with respect to HS (10.3 ± 7.7; range 0–36; *p* < 0.001); this difference remained significant when assessing anterior or posterior chest area (*p* < 0.001), but not when evaluating the lateral chest areas alone or when comparing either the AS or the PsA group with HSs. No significant differences were found between the PsA and AS groups (19.9 ± 11.2; range: 0–51 and 20 ± 10.6; range: 2–46, respectively). The posterior part of the chest showed a higher PLI score than the anterior and lateral parts (with the latter being significantly lower than the posterior PLI score) (Figure 2). PLI scores from individual areas (anterior, posterior or lateral chest) correlated with the mean PLI score (*p* < 0.05 for all).Therefore, we decided to use the mean value to evaluate correlations between LUS and other parameters.

### 3.2. PLI Score Correlation with Demographic Data and PROs

We did not find any significant correlation between PLI score and sex, BMI (significantly higher in males) or age at the time of the study.

The prevalence of smokers was not significantly different between patients and HSs (35.6% vs. 35.7%). A positive correlation between PLI score and smoking status (yes/no), including both former and current smokers (*p* < 0.003), as well as between PLI score and number of pack/years (*p* < 0.05) was observed in both SpA patients and HSs. In both patients and HSs, the anterior chest region showed the strongest correlation with smoking status (both for presence/absence and pack/year). A positive correlation was also noted between dyspnea (assessed using mMRC) and smoking status (*p* = 0.05), with an even stronger correlation noted with the number of pack/year (*p* = 0.019).

The mean PLI score was not correlated with PROs (LCQ, SGRQ, mMRC and SF-36); the only positive correlation was observed between the mMRC score and anterior chest PLI (*p* = 0.05), as well as with the SF-36 score. By contrast, PROs were correlated with each other (showing a good concordance for absence/presence of symptoms; *p* = 0.001; r = 0.500).

### 3.3. PLI Score Correlation with Clinical Data

The presence of crackles (even if in only 10 patients) was significantly related to the PLI score (*p* < 0.003).

Disease activity, disease duration, age at disease onset (even when considering onset <40 years, onset between 40 and 65 years and onset >65 years groups) or the presence of psoriasis were not related to PLI total score. Disease activity in the AS group was significantly correlated to SGRQ single domains (activity and impact; *p* < 0.05) and with all domains of SF-36 (*p* < 0.05).

### 3.4. PLI Score Correlation with Treatments

**Figure 3:**
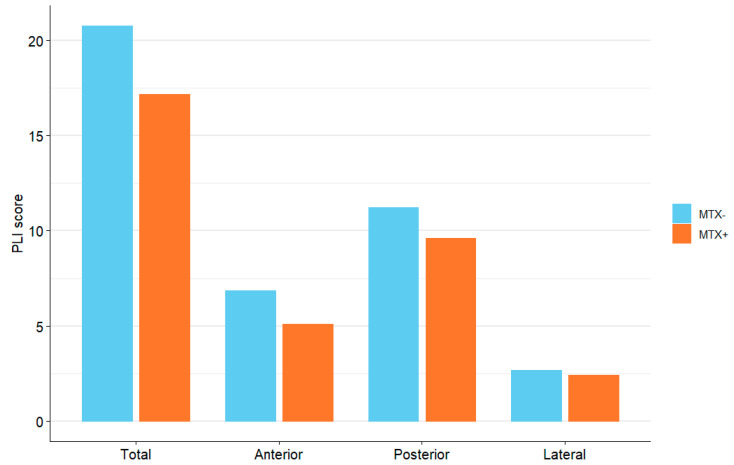
Mean PLI score in different chest areas, according to MTX exposure.

We did not observe any significant correlation between bDMARDs or csDMARDs use and the PLI score. When evaluating single treatments, we found a significant relationship between IL17 inhibitors and PLI score (*p* < 0.0065 vs. no bDMARDs) and a numerical difference in favor of TNFa inhibitors vs. no bDMARDs (18.4 and 21.7, respectively). When considering only MTX use, patients treated with MTX showed a numerically lower PLI score (for both total and individual areas) (Figure 3).

## 4. Discussion

ILD represents one of the most severe complications in different rheumatic diseases (SSc, RA, other connective tissue diseases and vasculitis), being the first cause of mortality. ILD prevalence varies according to the method used to detect it, ranging between 10 and 60% (i.e., in RA it is between 10–70%) [53]. Little is known about the real prevalence of ILD in SpA: few data are available on AS, with some studies reporting that about 60% of AS show different degrees of lung disease (pleural thickening in 18% and ground-glass or interlobular thickening in about 11% of the patients), supporting the need for an early screening of patients [6]. Literature data in PsA are scarce and suggest that lung involvement is not common [11,13,14], with nodular or ground glass in the upper lobes and honeycombing in the lower lobes [12].

The gold-standard imaging technique for ILD assessment is HRCT (usually using Warrick’s score). The radiation, necessary to complete a HRCT impose the need for other “less invasive” imaging techniques to be used for large screening programs. Lung ultrasound, a “less invasive”, easy-to-perform and cost-effective technique, has recently shown potential for ILD evaluation by detecting two findings: B-lines and pleural irregularity. Those findings have demonstrated a direct correlation with HRCT score both in cardiology, pneumology and, more recently, rheumatology [6,18,32].

LUS standardization is still pending, and OMERACT is currently working on its development. Most of the papers in the literature focus on B-lines using different scanning protocols (with ranges from 72 to 10 intercostal spaces) [20,21]. Fewer papers have evaluated the pleural line, both in terms of thickening (i.e., a thickening of more than 2.8 mm in at least two contiguous areas correlate with ILD on HRCT) [21,23] and pleural irregularity. Pinal Fernandez et al. demonstrated a numerically higher sensitivity and specificity for pleural line irregularity with respect to B-lines [24]. Pleural line irregularity (in terms of fragmentation, thickening, interruption, or irregularity) has not been demonstrated in healthy subjects or in patients with congestive heart disease, apparently providing more specific results for ILD [25]. A similar result was demonstrated in a population of SSc patients, where B-lines were positive in both patients with and without ILD, while pleural line irregularity was present only on those presenting ILD [22]. It is well known that isolated B-lines can be observed in the lower lung regions of healthy subjects; however, this has not yet been described for PLI. Moreover, applying the same scanning protocol to the control group helps minimize variations due to anatomical or physical factors. Given all those points, we decided to use pleural line irregularity evaluation and not B-lines because we consider its evaluation easier and faster than counting B-lines (especially if multiple). Feasibility is indeed really important, especially if someone wants to apply LUS on a large number of patients (such as those with SpA) on a regular basis, during the usual visiting time.

In the present work, we aimed at examining a sample of our SpA cohort to understand if LUS could be a useful screening test for ILD to be used in asymptomatic subjects.

As reported in Table 1, our SpA patients have a relatively long disease duration (15.5 yrs) and are relatively older (57.8 yrs). This population may not be ideal for applying our imaging screening tool, as screening younger patients with shorter disease duration could be more informative for guiding therapy decisions. However, due to the lack of existing data, we conducted a cross-sectional study to evaluate the feasibility and potential utility of this approach.

We decided to use the Pinal–Fernandez [24] scanning protocol and scoring system for the evaluation of pleural line irregularity. We made a separate evaluation for distinct areas (anterior, posterior and lateral), and the posterior part of the chest showed a higher PLI score than the anterior and lateral one (with the latter being significantly lower than the posterior PLI score), but no significant difference in their correlation with other clinical data was shown. Our data demonstrate a significant higher pleural line irregularity score in SpA patients (19.9 in SpA group vs. 10.3 in HS).

Recently, IL17 has been proposed as a pro-inflammatory mediator (similar to TNFα and TGF-β) involved in ILD development, as demonstrated in rat models, where both IL17 and TNFα (together with other cytokines) were increased (after bleomycin induction of ILD) in the lung tissue samples after hematoxylin-eosin staining [54]. The possible role of IL-17 in ILD is highlighted in systemic sclerosis patients by significantly elevated IL-17A levels in the patients with ILD compared to controls [55]. Additionally, a positive effect on the fibrotic changes of lung tissues and improved mechanical pulmonary functions in bleomycin-induced pulmonary fibrosis were shown during the suppression of IL-17 production (through the inhibition of cytokines controlling Th17 differentiation) with theophylline [56]. Therefore, IL17/23 pathway inhibitors could have potential anti-fibrotic effects, as shown in some patients [8]. All those preliminary data suggest the need for large-scale studies in population of patients treated with IL17/23 pathway inhibitors, possibly using HRCT for assessing ILD. A possible link between the use of anti-TNF drugs and the presence or progression of ILD was hypothesized, and recent evidence has suggested the apparent non-efficacy of TNFα inhibitors in controlling/reducing ILD progression with respect to other treatments (i.e., abatacept, rituximab) [57,58]. Although these findings are intriguing, we must acknowledge that our study, due to the small sample size, cannot provide definitive evidence supporting or refuting them. We performed exploratory sub-analyses comparing treatments within our cohort; however, the data presented in the Results section should be interpreted with caution and not regarded as statistically significant, despite being consistent with recent literature. For example, MTX was confirmed not to be a risk factor for ILD in a large cohort study by Provan et al. [14], as reported by other Authors [59,60,61]. Similarly, in our limited sample, patients treated with MTX showed lower ILD scores compared to those who did not receive MTX.

Another limitation in our study is represented by the fact that we only had data on exposure and not on duration of the treatments. This could have potentially affected the results. Prospective and larger studies are needed to better evaluate the impact of different treatments on ILD development.

We did not find any correlation between disease duration and pleural line irregularity, confirming the results already reported in the literature for HRCT [6].

The absence of a correlation between PROs (LCQ and SGRQ) and pleural line irregularity scores, as well as between SF-36 and LUS, reflects the possible subclinical phase of ILD in our patients (only a few of them exhibited a score of 2 in any region). This means that PROs cannot be used as a screening tool to detect a very early phase of ILD in SpA patients.

We did not notice any significant difference in pleural line irregularity score between AS and PsA, so we could speculate that results already known about ILD in AS can also be applied to PsA.

A positive trend in favor of a correlation between pleural line irregularity score and age was observed, warranting further study.

A significant correlation was found between smoking habits and LUS results (both for actual or previous smoking and for pack/years). The prevalence of smokers was not significantly different between the SpA group and the HSs (35.6% vs. 35.7%), and a significant positive relationship between smoking habits and PLI score was observed in both patients and HSs (*p <* 0.003). The strongest correlation between different areas of the lung and PLI irregularity was observed, in both the groups, in the anterior chest. Even if we do not have specific data on the impact of smoking on pleural line changes, it is well known that smoking represents one of the major risk factors for the development of ILD [62]. We also found a clear significant correlation between smoking habits and mMRC in terms of the presence of dyspnea (*p =* 0.05) and pack/year (*p* = 0.019).

The weakest point of our study is the low number of patients enrolled (27 AS and 46 PsA), which is not sufficient to fully explore a low-prevalence condition such as symptomatic ILD in SpA. In addition, HRCT was not used as the gold standard comparator because, as both our patients and control subjects were asymptomatic, there was no clinical indication to request it. Finally, it would not have been ethical to perform HRCT examination only for scientific purposes. Therefore, we could not calculate any PLI cut-off results in our population to discriminate between ILD-positive and ILD-negative patients. We only investigated the general prevalence of PLI in the population studied.

Nevertheless, as a sort of surrogate, we found a significant correlation between clinical examination findings (presence of crackles) and PLI score, thus confirming the link between the presence of structural changes (producing crackles) and LUS findings.

### Future Research Directions

In light of the general results we obtained, our study serves as a proof of concept that demonstrates a possible high prevalence of asymptomatic ILD in SpA patients, suggesting the need of studies on larger groups to confirm our data. Moreover, prospective studies are needed to understand if LUS ILD will ever become symptomatic. Up to now, the high prevalence of symptomatic ILD in RA, a form of chronic arthritis somehow similar to PsA, is well established.

LUS proved to be feasible, non-invasive and potentially useful for screening larger groups of patients. According to the good reliability observed between the two trained sonographers, multicenter studies might be possible.

Up to now, we still need a standardization of the technique (i.e., number of intercostal spaces to study, scoring system, etc.) and more data on the applicability of LUS for screening purposes and, even more, for the follow-up of patients. OMERACT is working on this topic [27].

Anyway, based on the limited knowledge we have now, we still recommend confirming a LUS positive result with more standardized techniques (i.e., pulmonary function tests) before referring the patient for HRCT, which remains the gold standard for a complete assessment of ILD, providing important information on deeper lung structural changes and allowing for the differentiation of specific ILD patterns (i.e., NSIP or UIP).

## 5. Conclusions

This is the first study assessing ILD in SpA patients using LUS. Our findings indicate a higher amount of pleural line irregularity in SpA patients compared to HS. Therefore, the true frequency of ILD in SpA may be underestimated. Larger, preferably prospective, studies are needed to better define the clinical value of PLI assessment in this population, as well as to clarify the prevalence and clinical significance of ILD in SpA patients. Smoking habit was the only clinical feature correlated with pleural line irregularity on LUS examination in our population. Future application of LUS could be better addressed at the beginning of the disease and then repeated on a regular basis, to achieve real personalization of SpA treatment according to the early recognition of ILD.

## Figures and Tables

**Table 1:** Demographic and clinical characteristics of subjects enrolled in the study.

	PsA + AS (n = 73)	HS (n = 56)	PsA (n = 46)	AS (n = 27)
M	38 (52.1%)	30 (53.6%)	25 (54.4%)	14 (51.9%)
F	35 (47.9%)	26 (46.4%)	21 (45.6%)	13 (48.1%)
Age (±SD)	57.8 (11.9)	56 (15.6)	58.8 (12.3)	56.1 (11.4)
BMI (±SD)	26.2 (4)	24.4 (2.8)	26.8 (4.1)	24.7 (3.5)
Smoke habits (%)	26 (35.6%)	20 (35.7%)	15 (32.6%)	11 (40.7%)
Disease duration, yrs (±SD)	15.5 (11.5)	-------	14.9 (10.6)	16.4 (13.5)
ASDAS (±SD)	-------	-------	-------	2.2 (1.4)
DAPSA (±SD)	-------	-------	14.1 (10.5)	-------
Psoriasis	42	-------	42	0
TNFa inhibitors, N (%)	10 (13.7)	-------	23 (50)	22 (81.5)
Other bDMARDs, N (%)	10 (13.7)	-------	9 (19.6)	1 (3.7%)
csDMARDs (all), N (%)	30 (41.1)	-------	23 (50)	7 (25.9)
csDMARDs (MTX), N (% csDMARDs)	18 (60)	-------	14 (60.1)	4 (57.1)

## Data Availability

The original contributions presented in this study are included in the article. Further inquiries can be directed to the corresponding author(s).
